# Gaussian-Based Smooth Dielectric Function: A Surface-Free Approach for Modeling Macromolecular Binding in Solvents

**DOI:** 10.3389/fmolb.2018.00025

**Published:** 2018-03-27

**Authors:** Arghya Chakravorty, Zhe Jia, Yunhui Peng, Nayere Tajielyato, Lisi Wang, Emil Alexov

**Affiliations:** ^1^Computational Biophysics and Bioinformatics, Department of Physics and Astronomy, Clemson University, Clemson, SC, United States; ^2^Department of Chemistry, Clemson University, Clemson, SC, United States

**Keywords:** Gaussian-based dielectric function, binding, macromolecular interactions, Poisson-Boltzmann equation, macromolecular solvation, surface free

## Abstract

Conventional modeling techniques to model macromolecular solvation and its effect on binding in the framework of Poisson-Boltzmann based implicit solvent models make use of a geometrically defined surface to depict the separation of macromolecular interior (low dielectric constant) from the solvent phase (high dielectric constant). Though this simplification saves time and computational resources without significantly compromising the accuracy of free energy calculations, it bypasses some of the key physio-chemical properties of the solute-solvent interface, e.g., the altered flexibility of water molecules and that of side chains at the interface, which results in dielectric properties different from both bulk water and macromolecular interior, respectively. Here we present a Gaussian-based smooth dielectric model, an inhomogeneous dielectric distribution model that mimics the effect of macromolecular flexibility and captures the altered properties of surface bound water molecules. Thus, the model delivers a smooth transition of dielectric properties from the macromolecular interior to the solvent phase, eliminating any unphysical surface separating the two phases. Using various examples of macromolecular binding, we demonstrate its utility and illustrate the comparison with the conventional 2-dielectric model. We also showcase some additional abilities of this model, viz. to account for the effect of electrolytes in the solution and to render the distribution profile of water across a lipid membrane.

All biological macromolecules (proteins, DNAs, RNAs) perform their functions in cellular or body liquids, which are predominantly aqueous. When taken out of the water phase and placed in a different environment such as vacuum, air, alcohol etc., these macromolecules are almost always rendered dysfunctional (Arteca et al., 2001). Even more so, the alterations of native water phase characteristics such as pH, salt concentration and presence of other molecules, can also cause complete unfolding and abolishment of macromolecular interactions (Alexov, 2004; Talley and Alexov, 2010; Onufriev and Alexov, 2013; Petukh et al., 2013; Spinozzi et al., 2016). These facts reflect the importance of the presence of the water phase for the native functionality of macromolecules of which macromolecular recognition is certainly a significant part. Therefore, when studying macromolecular binding, any model of macromolecular interaction should account for the presence of water and its effects on the process of binding.

From the point of view of modeling water phase, the computational protocols can be broadly classified into explicit and implicit (Reddy et al., 2014; Li et al., 2015). In the explicit protocol, water and the macromolecules are presented with atomistic level of details; this avoids the necessity of defining a macromolecule-water boundary. As part of an implicit protocol, water phase is treated as a continuum dielectric medium. But in addition to losing some important atomistic details, this protocol also require macromolecule-water boundary to be defined.

By the conventional protocol of studying macromolecular binding, the 3D structure of the macromolecular complex (referred to as a bound state) and its monomers (together referred to as unbound state) is solvated separately, their respective solvation free energies are computed and they are subtracted to obtain the solvation component of the effective binding free energy (Gilson and Zhou, 2007; Aldeghi et al., 2017; Mobley and Gilson, 2017). In doing so, the macromolecule-water interactions are naturally rendered different in the bound and unbound states, since there are parts of the monomer-monomer interface which are buried in the bound state but in the unbound state, they are exposed to water. Capturing its effect on the macromolecular binding, therefore, requires an appropriate representation of the macromolecule-water border.

The most commonly used definitions of the macromolecule-water boundary are the solvent accessible surface (SAS) and the solvent excluded surface (SES), which is also well known as the molecular surface (MS) (Decherchi et al., 2013). Other surface definitions include van der Waals (VDW) surface, Gaussian surface, spline surface, geometric flow surface, blobby and skin surfaces (Li et al., 2014). These surfaces are constructed purely based on geometric description of the solute and solvent atoms and consequently their differences are geometric. Nevertheless, they all introduce a sharp border between the macromolecule and the surrounding water phase. This results in an abrupt and unphysical dielectric jump in continuum dielectric models. Modeling protocols that combine molecular dynamics (MD) simulations with Poisson-Boltzmann (PB) modeling of solvation cause any small change in the macromolecular conformation to alter the dielectric border between macromolecule(s) and water phase (Wen et al., 2004; Wang et al., 2010, 2017; Cai et al., 2011; Geng and Wei, 2011). Such definitions overlook the physical nature of interactions between macromolecule and water and the ability of water molecules to mediate binding based on its location around the macromolecule (e.g., Ikura et al., 2004). This also overlooks the fact that the hydrophobic surface patches or cavities are naturally not very hydrated, while the hydrophilic patches are (Barnes et al., 2017; Yang et al., 2017; Shin and Willard, 2018). Therefore, a physically sound protocol that delivers a MS should not only account for the geometry but also consider the physio-chemical properties of a macromolecular surface.

Recently, the matched interface and boundary (MIB) method was introduced (Zhao and Wei, 2009; Xia et al., 2011). The method rigorously enforces the solution and flux continuity conditions at the biomolecule-solvent dielectric (Chen et al., 2011; Xia et al., 2014). Similarly, the variational implicit solvent method (VISM) was proposed to account for differential hydration depending on the physicochemical and structural characteristics of the biomolecule (Cheng et al., 2009). It uses an effective solvation free-energy function that depends solely on the position of solute-solvent interface and solute atoms. It couples several energy terms such as the volume and interface energies of solutes, the solute-solvent VDW interaction energy and the solute-solute mechanical interactions energy (Zhou et al., 2014; Sun et al., 2015; Ricci et al., 2017). In addition, a curvature dependent surface tension is incorporated to account for the different hydration of concave and convex surfaces.

Besides accounting for the physio-chemical properties of the macromolecule-water interface, it is equally important to consider that macromolecules do not stay “frozen” in their environment. Molecular flexibility continuously updates local interactions of solvent-exposed atoms with the solvent and other solute atoms. Inspired by these challenges, we recently developed a solvation model known as a Gaussian-based smooth dielectric distribution model (Li et al., 2013), to mimic the abovementioned phenomenon in continuum electrostatics. In addition, this model was shown to capture physio-chemical properties as well by assigning a lower dielectric to hydrophobic residues and a higher dielectric to hydrophilic ones (Li et al., 2013).

In the Gaussian-based dielectric model, the continuum solvent/water medium (identified by a larger dielectric constant) is smoothly fused with the macromolecular region (that has a lower dielectric constant). It ensures that a smooth transition of the dielectric properties occurs from the macromolecular interior to the water phase and subsequently, eliminates the need of a MS; a sharp border between the macromolecule and water. The idea is to represent each atom as an atom-centered Gaussian density function (Equation 1) as opposed to a hard sphere (Grant et al., 2001). The resulting total atomistic density (Equation 2) is then transformed into a 3D distribution of dielectric “constant” throughout the entire modeling space (Equation 3). Thus, densely-packed atoms result in region of space that will have low dielectric value, while loosely-packed space regions, such as MS, will have high dielectric constant. The motivation is to mimic flexibility via dielectric constant, since it is expected that loosely packed space regions will allow for larger flexibility than the highly packed regions.

(1) $$\begin{array}{l} \rho_{i}\left(\vec{r}\right)=\exp\left(-\frac{{\left(\vec{r}-\vec{r_{i}}\right)}^{2}}{\sigma^{2}R_{i}^{2}}\right) \end{array}$$

(2) $$\begin{array}{l} \rho_{mol}\left(\vec{r}\right)=\;1-\prod_{i}(1-\rho_{i}\left(\vec{r}\right)) \end{array}$$

(3) $$\begin{array}{l} \epsilon \left(\vec{r}\right)=\rho_{mol}\left(\vec{r}\right)\epsilon_{in}+\left(1-\rho_{mol}\left(\vec{r}\right)\right)\epsilon_{out} \end{array}$$

For an atom “i” centered at $\vec{r_{i}}\;$ and for a 3D point $\vec{r}$, quantities ρ_*i*_, ρ_*mol*_, and $\epsilon \left(\vec{r}\right)$ denote individual atomic probability, the collective probability and the dielectric distribution in space, respectively. The basis for our model is also the basis of solvation models applicable to Molecular Dynamics (Gallicchio and Levy, 2004; Grant et al., 2007). A reference solute internal dielectric constant (ϵ_*in*_) and the solvent dielectric constant (ϵ_*out*_) are used to transform atomic densities to a dielectric distribution (technical details of implementation in Delphi are elsewhere Li et al., 2013. To reduce computational time, the contribution of neighboring atoms at each grid midpoint is truncated at distance 3σ). Parameter σ, the spread of atomic density, is assigned an optimal value of 0.93 obtained from an empirical study meant to yield experimental hydration free energies of small molecules (Li et al., 2013, 2014) and pKa's of protein titratable residues (Wang et al., 2015) using the Gaussian model (see Table S1).

The result is a smooth Gaussian-based dielectric function throughout the entire computational space. The necessity of such an approach is evident from the other works (Wang et al., 2001; Sinha et al., 2008) which show that the water molecules in the proximity of the macromolecule and in its cavities have different dielectric responses from those far out in the bulk region. Moreover, an inhomogeneous dielectric distribution in the region between the molecules also highlight how the long-range electrostatic interactions are affected in the process of recognition before binding (Li et al., 2017).

The Gaussian-based smooth dielectric model has been implemented in Delphi (Li et al., 2012). In the paragraphs to follow, we cover some of the important examples of macromolecular binding and illustrate how the Gaussian-based dielectric model delivers a physically realistic picture of macromolecular interaction in water, both qualitatively and quantitatively. We extend the example of its usage to highlight its ability to incur the effect of salt/electrolyte-ions in the solution as well as its relevance to the distribution of water molecules around lipid bilayers.

## Protein-protein interactions (barnsase-barstar)

The barnase-barstar complex from *Bacillus amyloliquefaciens*, where Barnase (Bn) is an extracellular ribonuclease and Barstar (Bs) is its intracellular inhibitor, has been used extensively in studies (Hartley, 1989; Janin, 1997; Hoefling and Gottschalk, 2010). An experimental study of their water mediated-interaction has reported that the water molecules (H_2_O) crystallized at the Bn-Bs interface have different B-factors (Ikura et al., 2004). The different B-factors have been attributed to the number of H-bonds these water molecules made with either or both monomers and their ability, henceforth, to reorient and respond to local electrostatic field.

**Figure 1:**
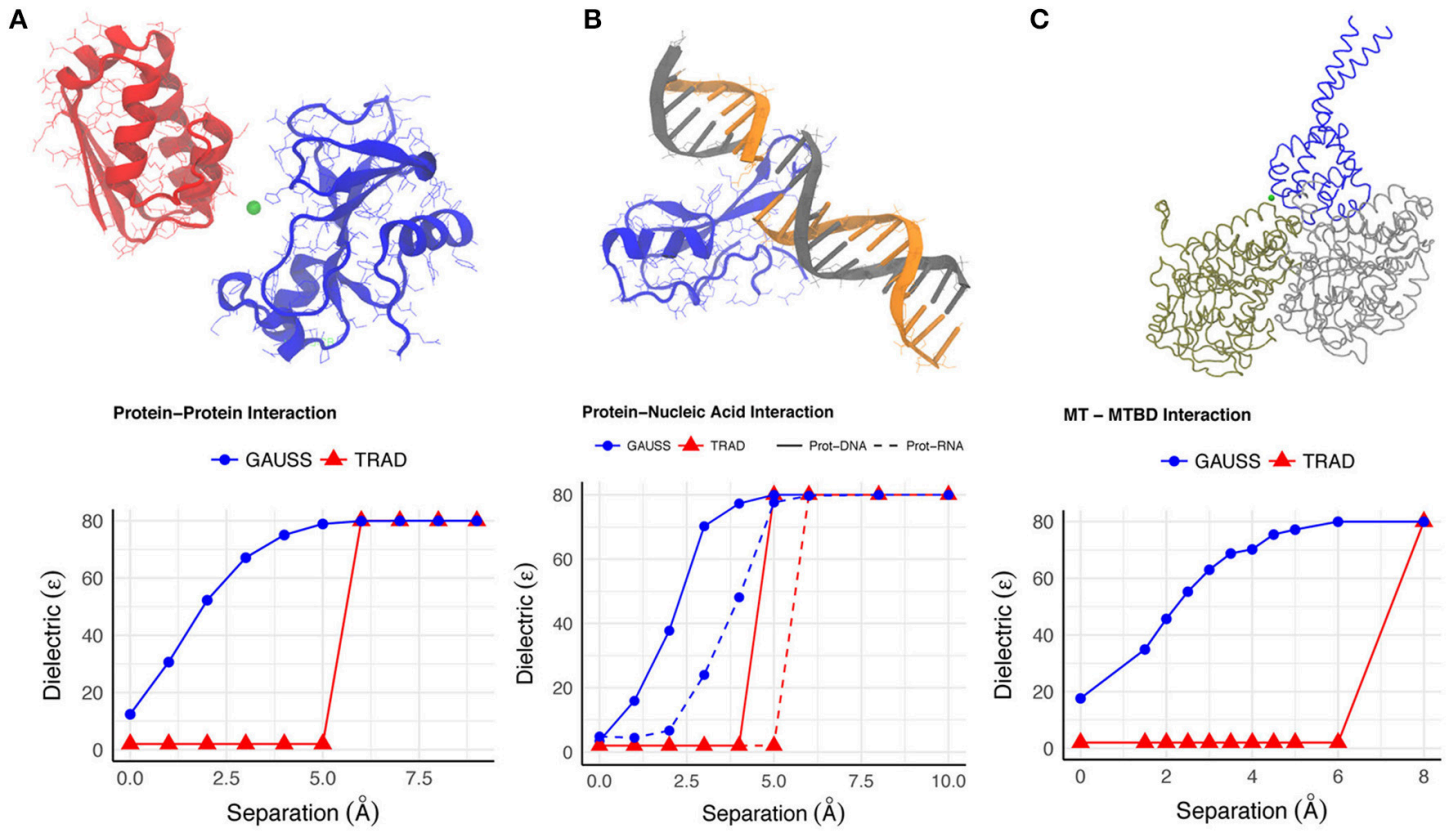
The variation of the dielectric constant **(A–C)** at a point between the interfaces of binding molecules in each of the example cases is shown as a function of the distance by which the molecules were separated. The point where dielectric constant was calculated is shown with green dot in the figures.

**Figure 2:**
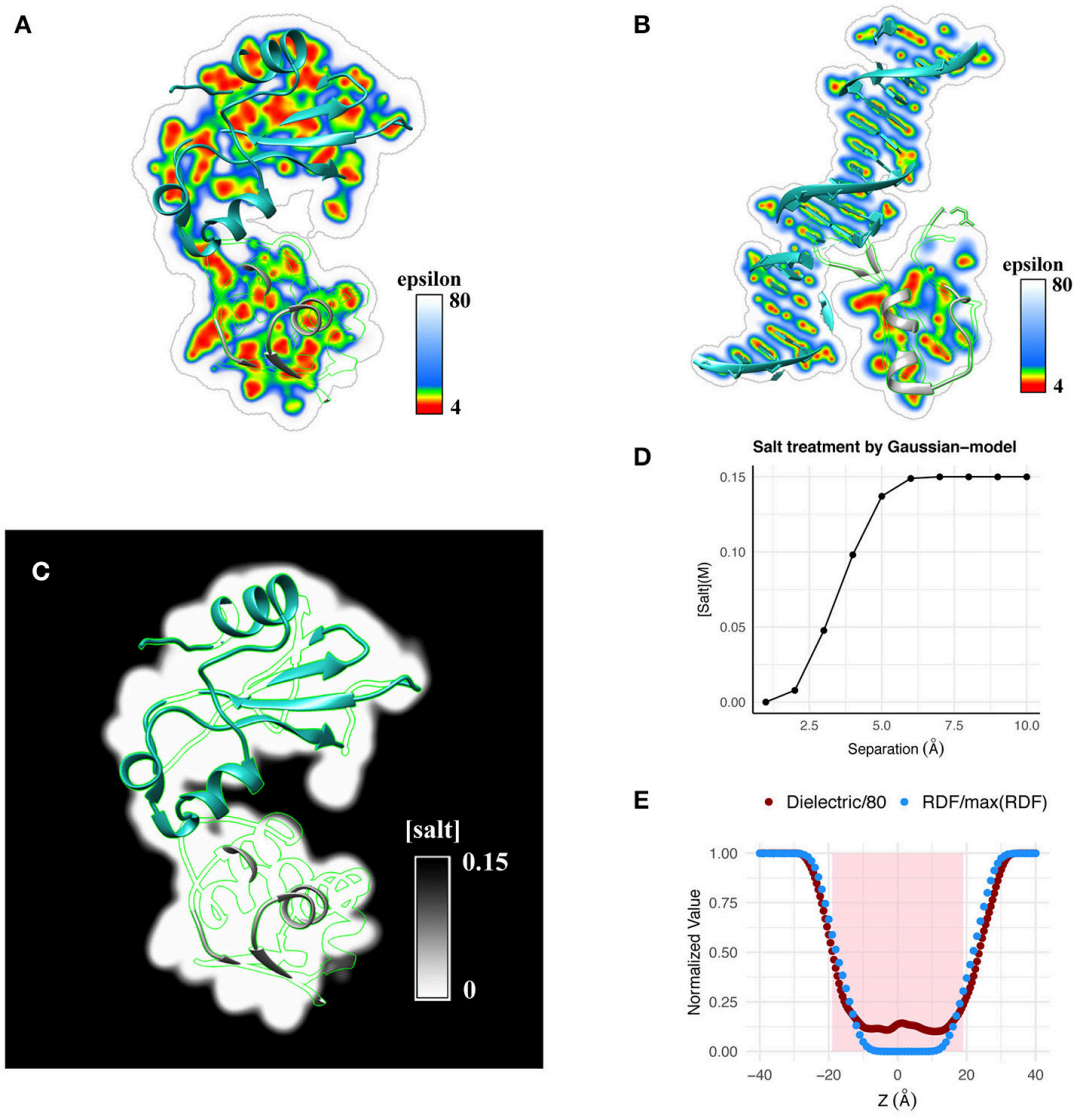
Qualitative illustration of dielectric distribution for **(A)** a protein-protein complex and **(B)** a protein-DNA complex obtained using the Gaussian-based dielectric distribution model is shown. **(C)** Salt distribution around the same protein-protein complex obtained using the salt model implemented in Gaussian-based dielectric model is shown. **(D)** Variation of salt concentration using the same model is shown at a point at the interface of Barnase-Barstar as a function of their separation distance. **(E)** Normalized radial distribution (RDF, normalized by the maximum RDF value) of explicit waters across a POPC membrane is compared with the dielectric distribution (normalized by 80, i.e., Dielectric/80) obtained with the Gaussian-based dielectric model across the thickness of the membrane.

We use the Gaussian-based dielectric model (GAUSS) to provide a description of the dielectric constant distribution at the interface of Bn-Bs complex (PDB: 1X1X) as we move its monomers apart in space. For comparison, we do the same with the *traditional 2-dielectric* model (TRAD). The results are shown in Figure 1A for configurations where the monomer centers are moved apart by distances in the range of 0–10Å. Figure 2A shows a qualitative description of the dielectric distribution around the complex (bound state) using Gaussian-based smooth dielectric function.

One can appreciate the lack of sharp change in the dielectric achieved with the Gaussian model, suggesting a smooth change of dielectric constant value in the space between the monomers as they are moved apart. Even at very low separations, the space between the Bn and Bs exhibits a dielectric between ϵ_*in*_ and ϵ_*out*_ but not identical to ϵ_*in*_ (Figure 1A). Such a trend depicts how the space between interfaces begin to gain higher dielectric values mimicking the increased flexibility of interfacial residues upon separation and increased probability of water molecules to enter there. This also resonates with the observation that the interfacial water molecules, when there is very little room between interfaces, have different mobility compared to the bulk water due to plausible interactions with the monomers.

## Protein-nucleic acids interaction

The interactions between protein and nucleic acid is equally important as those amongst proteins and they play crucial roles in various cellular functionalities such as transcription, translation, replication, repair and rearrangement of nucleic acids. Elucidation of the mechanism in protein-nucleic acid interactions and further prediction of the important properties have been major goals in some past studies (Lejeune et al., 2005; Rohs et al., 2010). Below, we use two examples to show the smooth transition of the dielectric properties in the protein-nucleic complexes and the advantages of Gaussian-based smooth dielectric function to mimic the change of the dielectric properties of the space between interfaces.

One example is the structure of human MeCP2 Methyl-CpG binding domain in complex with Methylated DNA (PDB: 3C2I) and the other example is the structure of *Bacillus Anthracis* ribosomal protein S8 in complex with an RNA aptamer (PDB:4PDB). The protein and DNA/RNA were moved apart along the line connecting their respective geometric centers by distances 0–10 Å and the average dielectric at the interface was likewise calculated (using both dielectric distribution methods). This variation distance is plotted in Figure 1B. From a physical perspective, one expects that the average dielectric at the interface will undergo a gradual change as the protein-DNA/RNA (un)binds. Thus, the averaged dielectric in the completely unbound state is 80 (distance of separation ≥6Å), revealing that the corresponding area is entirely consumed by the water phase which exhibits bulk properties. More interesting is the partial bound state, where the average dielectric lies between that of ϵ_*in*_ and ϵ_*out*_. The Gaussian-based dielectric model captures such an expectation resulting in a smooth transition from bound to unbound states. However, the traditional 2-dielectric model provides non-realistic picture with a sharp dielectric jump at about 5Å separation of monomers. The 3D distribution of dielectric constant in bound state of protein-DNA is shown in Figure 2B. Similar to the protein-protein complex, the dielectric constant at the interface varies a lot from values similar to macromolecular interior to those approaching the dielectric of bulk water.

## Protein-microtubule interaction (microtubule-binding-domain(MTBD)-microtubule)

The cytoskeleton has an important role in cellular activity like cell division, cell movement and helping the cells maintain their shape and internal organization. A principle component of cytoskeleton is a microtubule (MT) which is like a rigid hollow rod (~25 nm in diameter) populating the cell interior. Molecules and cargo containing-vesicles or organelles are carried on the microtubules around the cell by motor proteins which are powered by adenosine triphosphate (ATP).

Here, we have considered a large segment of MT bound to the binding domain (MTBD) of cytoplasmic dynein, a motor protein. The MTBD was moved away from the original position along axis perpendicular to the MT by 0–10 Å and the average dielectric value around the midpoint between these two proteins, was calculated. The calculations were made using the Gaussian-based as well the traditional 2-dielectric model.

Figure 1C shows that at the interface in the bound state, the average dielectric constant is close to the internal reference dielectric constant for the proteins (ϵ_*in*_). As the MTBD is moved away, the dielectric value rendered by the Gaussian-based model increases and eventually saturates at ϵ_*out*_ (80 here). This transition reflects the ability of the water molecules flooding the void between MTBD and MT to behave differently than those in the bulk because of their interaction with interfacial residues. This example not only corroborates the observations from the Barnase-Barstar complex, but it also demonstrates the ability of the Gaussian-based model and Delphi to work on very large systems.

## Salt-distribution in the solvent phase modeled using Gaussian-based model

The advantages of Gaussian-based model can be demonstrated via modeling of salt concentration as well. The presence of ions or salt in PB solvation models is accounted for by their Boltzmann distribution, i.e., their concentration in the solvent phase is proportional to the Boltzmann factor corresponding to the electrostatic energy of an ion at some point in the solvent region. The surface-free nature of the Gaussian-based dielectric model eliminates the provision of a clearly demarcated solvent region which therefore, challenges its ability to incorporate the non-trivial effects of salt on binding (Zhou, 2001; Bertonati et al., 2007). This issue has been investigated and solved in our recent work (Jia et al., 2017). Our solution to this problem was inspired by the fact that charges, which migrate to regions with different dielectric constants, sustain a (de)-solvation energy or a “penalty”. In our Gaussian-based model, this penalty is expressed using Born's formalism where an ion transferring from bulk solvent to regions of lower dielectric incurs a penalty (in SI units):

(4) $$\begin{array}{l} \Delta G_{penalty}(\vec{r})=-\frac{N_{A}z^{2}e^{2}}{8\pi \epsilon_{0}r_{0}}\left(\frac{1}{\epsilon (\vec{r})}-\frac{1}{\epsilon_{out}}\right) \end{array}$$

Here *N*_*A*_–Avogadro constant, *e*–elementary charge and $\epsilon \left(\vec{r}\right)$–space-dependent dielectric as calculated by Gaussian-based model. The penalty term influences an ion's ability (of charge *q*_*i*_ = *z*_*i*_*e*) to be present at some $\vec{r}$ in the solvent medium which when added to the electrostatic potential there ($-{\text{q}}_{\text{i}}\varphi \left(\vec{\text{r}}\right)$) renders the following expression for PBE:

(5) $$\begin{array}{l} \vec{\nabla }\cdot \left[\epsilon \left(\vec{r}\right)\nabla \varphi \left(\vec{r}\right)\right]=-4\pi (\rho_{solute}\left(\vec{r}\right)\\ +\sum_{i=1}^{N}q_{i}c^{bulk}exp\left(\frac{-q_{i}\varphi \left(\vec{r}\right)-\Delta G_{penalty}(\vec{r})}{RT}\right)) \end{array}$$

Quantities $\varphi \left(\vec{r}\right)$, and $\rho_{solute}\left(\vec{r}\right)$ are the electrostatic potential and charge density of a solute at $\vec{r}$, respectively; *c*^*bulk*^ is the bulk ion concentration and *T* is the temperature.

To illustrate the usage of Gaussian-based smooth dielectric model in computing salt concentration distribution, we examine a protein-protein complex. Qualitatively, this is shown in Figure 2C. Quantitatively, salt distribution at the midpoint of Barnase-Barstar complex as a function of their separation is shown in Figure 2D. It can be seen that ions can propagate inside the binding interface if there are small cavities allowing for transient ions to come in.

## Predicting water distribution across lipid bilayers using Gaussian-based dielectric model

Here we demonstrate that Gaussian-based dielectric model can mimic the effect of water molecules penetrating inside biological macromolecules. Lipid bilayer membranes in animal cells are exposed to the extra-intracellular fluids, which are aqueous electrolyte solutions. These membranes sustain very high hydrostatic and osmotic pressures (as high as 18 KPa; Bereiter-Hahn, 2005) to preserve the shape of the cell and contain the cytoplasmic contents. Therefore, interaction, diffusion and permeation of water with and across lipid membranes are vital for osmoregulation and cell lysis. Subsequently, any lipid-water model should be appropriately represented for a computational study.

Using the Gaussian-based dielectric model, we show that the dielectric distribution across a lipid membrane matches well with the averaged distribution of water surrounding it; latter obtained from a 12 ns explicit water NPT-MD simulation of a POPC-lipid bilayer patch. The results are illustrated in Figure 2E. For better perspective, the values are normalized with respect to their respective maximum (e.g., 80 for dielectric constant). It can be seen that water molecules propagate inside the membrane resulting in a smooth profile from bulk water density to zero density in the core of the lipid bilayer. The dielectric constant profile replicates the trend by smoothly decreasing from 80 in the bulk phase to that of the membrane interior, i.e., the dielectric reaches the minimum possible value consistent with the zero density of water molecules. This finding provides additional support for our claim that the Gaussian-based dielectric function mimics the effect of water molecules near the macromolecular interfaces. The shaded region in Figure 2E is a crude representation of the membrane slab of thickness 38Å; the typical thickness of POPC membranes (Jo et al., 2009).

All the aforementioned examples and discussions have largely pertained to the ability of continuum models to model effect of water phase on macromolecular binding. We have demonstrated, both qualitatively and quantitatively, that a Gaussian-based smooth dielectric distribution provides a physically realistic picture of a solvated macromolecular system and consequently yields an appropriate representation of the environment around interacting/binding molecules. Our studies have reported its success not only in harnessing a more appealing description of solvation (Li et al., 2014) but have also shown that the model outperforms the conventional two dielectric model in predicting pKa's (Wang et al., 2015), optimum pH and proton transfer analysis (Peng and Alexov, 2017), predicting change in binding free energy upon mutation (Peng et al., 2017), etc. However, additional investigations are planned to test the performance of the model.

As the recent advances in solvation models continue to provide a more realistic picture of macromolecular behavior in water, efforts are also needed in developing time-inexpensive models for solvation and binding that can deliver experimentally measurable quantities. This is of importance because relevant experimental techniques deliver quantities that are ensemble averaged and are not merely pertinent to measurements made on a single molecule. At present, ensemble averaged quantities can be obtained by protocols like MM/PBSA (Srinivasan et al., 1998) and MM/GBSA (Kollman et al., 2000), which are rather time-consuming. Our Gaussian-based dielectric model, with its current abilities, has been shown to reproduce the ensemble average polar component of solvation energy from a single energy-minimized structure of a protein (Chakravorty et al., 2018). This paves way for future developments where the model can subsume more factors into account and deliver other energy terms. Our objective is to be able to use a single structure in conjunction with a Gaussian-based solvation model to yield experimentally verifiable free energies.

## Author contributions

AC, ZJ wrote the computational scripts for all analyses. AC, ZJ, YP, NT, LW, EA performed calculations and prepared the manuscript.

### Conflict of interest statement

The authors declare that the research was conducted in the absence of any commercial or financial relationships that could be construed as a potential conflict of interest.
