# Supplementary material for: Gaussian-Based Smooth Dielectric Function: A Surface-Free Approach for Modeling Macromolecular Binding in Solvents
Source: Front Mol Biosci. 2018 Mar 27;5:25. doi: 10.3389/fmolb.2018.00025 (PMC5881404; doi:10.3389/fmolb.2018.00025)
Supplement: Supplementary file 1 [file Table1.PDF]

Supplementary Material

Gaussian-based smooth dielectric function: A surface-free approach for modeling macromolecular binding in solvents

Arghya Chakravorty[1], Zhe Jia[1], Yunhui Peng[1], Nayere Tajielyato[1], Lisi Wang[2], Emil Alexov[1*]

[1]Computational Biophysics and Bioinformatics, Department of Physics and Astronomy, Clemson University, SC, USA
[2]Department of Chemistry, Clemson University, SC, USA

Table S1. Comparisons of performance of traditional 2-dielectric and Gaussian-based methods against experimental data and observable phenomena.

	Traditional 2-dielectric method	Gaussian-based dielectric method
pKa modeling: RMSD (calculated vs experimental) of 89 pKa's of SNase (J. Chem. Theory Comput. 2013, 9, 2126–2136)	2.43 pH units	1.77 pH units
Binding free energy changes upon 105 mutations (calculated vs experimental). (Bioinformatics. 2017 Oct 28. doi: 10.1093/bioinformatics/btx69)	0.62kcal/mol	0.54kcal/mol
Electron transfer in Bacterial Reaction Center: red – negative; blue - positive. Electron is supposed to be transferred from Qa→Qb. In traditional model, the electrostatic potential opposes the transfer, while in Gaussian-based model, it is almost neutral (Qa and Qb have similar potentials).	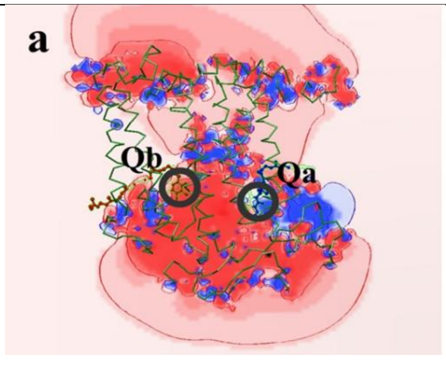	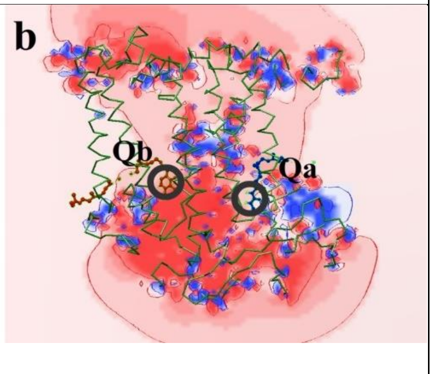
Linkage to the physical properties of amino acids ((J. Chem. Theory Comput. 2013, 9, 2126–2136).	Hydrophobic and hydropilic acids are assigned the same dielectric constant	Hydrophobic acids are assigned lower dielectric consatnt as compared with hydrophilic and polar residues
